# Supplementary material for: Effects of Pectinase Pre-Treatment on the Physicochemical Properties, Bioactive Compounds, and Volatile Components of Juices from Different Cultivars of Guava
Source: Foods. 2023 Jan 10;12(2):330. doi: 10.3390/foods12020330 (PMC9858270; doi:10.3390/foods12020330)
Supplement: Supplementary file 1 [file foods-12-00330-s001.zip › foods-2108825-supplementary.pdf]

Table S1. Effects of different cultivars and whether treated with pectinase or non-pectinase on the volatile components of guava juice

Types	Cultivar	Aldehydes		Alcohols		Esters		Terpenes		Ketones		Others		Total	
		N	RC (%)	N	RC (%)	N	RC (%)	N	RC (%)	N	RC (%)	N	RC (%)	N	RC (%)
Non-pectinase treatment	FR	8	52.84	7	14.60	7	16.20	14	11.88	1	3.76	3	0.72	40	100
	SF	8	60.91	5	18,47	5	14.18	9	4.98	0	0	3	1.47	30	100
	WR	5	12.14	5	4.19	0	0	23	83.09	2	0.58	0	0	35	100
	WP	5	18.78	7	14.80	0	0	24	65.71	1	0.49	1	0.23	38	100
Pectinase treatment	FR	9	31.56	9	13.12	10	35.69	13	13.02	1	5.24	4	1.36	45	100
	SF	11	47.54	8	9.92	9	26.72	13	14.72	1	0.22	3	0.88	45	100
	WR	7	42.26	5	6.63	2	0.96	20	47.82	2	1.69	2	0.47	38	100
	WP	7	27.37	6	8.00	2	1.32	21	61.33	3	1.53	1	0.46	40	100

N indicates number; RC indicates relative content.

Table S2. Effects of different cultivars and whether treated with pectinase or non-pectinase on the sensory attributes scores of guava juice

Types	Cultivar	Smell	Sweet and sour suitability	Taste	Color	Overall acceptability	Total
Non-pectinase treatment	FR	19.2	18.2	17.9	17.5	18.1	90.9
	SF	18.8	18.7	17.1	16.4	17.7	88.7
	WR	17.1	16.7	16.3	16.2	16.6	82.9
	WP	17.2	16.4	16.1	16.6	16.2	82.5
Pectinase treatment	FR	15.4	16.2	15.1	15.8	15.1	77.6
	SF	15.2	15.8	14.7	16	14.5	76.2
	WR	14.5	15.	14.2	15.5	15	74.5
	WP	14.3	14.9	14	17.9	14.9	76
